# Exposure to pro-tobacco and anti-tobacco media messages and events and smoking behaviour among adolescents in Gambia

**DOI:** 10.1186/s12889-024-18543-5

**Published:** 2024-04-15

**Authors:** Isaac Yeboah Addo, Evelyn Acquah, Samuel H. Nyarko, Kwamena S. Dickson, Ebenezer N. K. Boateng, Castro Ayebeng

**Affiliations:** 1 Concord Clinical School, University of Sydney, Sydney, Australia; 2 Centre for Social Research in Health, The University of New South Wales, Sydney, Australia; 3 Centre for Health Policy and Implementation Research, Institute of Health Research, University of Health, and Allied Sciences, Ho, Ghana; 4 Department of Epidemiology, Human Genetics & Environmental Sciences, School of Public Health, The University of Texas Health Science Center at Houston (UTHealth), Houston, Texas USA; 5 Department of Population and Health, University of Cape Coast, Cape Coast, Ghana; 6 Department of Geography and Regional Planning, University of Cape Coast, Cape Coast, Ghana; 7 Department of Research and Advocacy, Challenging Heights, Winneba, Ghana

**Keywords:** Tobacco use, Smoking, Significant others, Adolescent, Gambia, Mass media

## Abstract

**Background:**

Despite the widespread prevalence of adolescent smoking in Gambia, a West African country, there is limited research exploring the relationships between exposure to pro-tobacco and anti-tobacco media messages and events and smoking behaviour among young people. This study investigates the interplay of these exposures and smoking behaviour among 11-17-year-old adolescents in Gambia.

**Methods:**

Secondary data analysis was conducted using the 2017 Gambia Global Youth and Tobacco Survey (GYTS), which included a total of 9,127 respondents. Descriptive and inferential analyses, including proportions, Pearson’s chi-squared tests, and multivariable logistic regression models, were employed to estimate adjusted odds ratios (aOR) with 95% confidence intervals (CI).

**Results:**

The final model revealed significant associations between exposure to anti-tobacco media messages and events and smoking behaviour. Adolescents exposed to anti-tobacco media messages had a 29% increased odds of smoking (aOR 1.29,CI = 1.08,1.53) compared to those unexposed, while exposure to anti-tobacco media events showed a 31% increased odds (aOR 1.31,CI = 1.09,1.59) compared to those unexposed. Exposure to pro-tobacco messages, such as witnessing tobacco use on TV (aOR 1.41, CI = 1.17,1.69) and owning objects with tobacco brand logos (aOR 1.49,CI = 1.19,1.86), was associated with higher odds of smoking. Covariates, including sex, age, and exposure to smoking behaviour by significant others, also demonstrated associations with smoking behaviour. Notably, male respondents showed significantly higher odds of smoking (aOR = 4.01,CI = 3.28,4.89) compared to females. Respondents aged 15 years and older had increased odds of smoking (aOR = 1.47,CI = 1.22,1.76) compared to those below 15 years old. Those whose fathers smoke displayed higher odds of smoking (aOR = 1.35, CI = 1.04,1.76) compared to individuals with non-smoking parents. Additionally, those whose closest friends smoke showed remarkably higher odds of smoking (aOR = 2.87,CI = 2.37, 3.48) compared to those without such influence.

**Conclusion:**

This study underscores the significant impact of exposure to both anti-tobacco and pro-tobacco media messages and events on smoking behaviour among adolescents in Gambia. However, pro-tobacco messages had a greater influence on smoking prevalence than anti-tobacco messages and events. Understanding these associations is crucial for devising effective public health interventions aimed at reducing tobacco use in this population.

## Introduction

Tobacco use is a major contributor to morbidity and mortality worldwide, with most ‘chronic or heavy’ smokers initiating tobacco use during adolescence [1]. While smoking prevalence has reportedly declined in high-income countries over the last few decades, rates of tobacco use have increased in parts of sub-Saharan Africa (SSA) [1, 2]. Notably, smoking prevalence among youth and adolescents is high in many countries in the sub-region with over 20% of adolescents having reported using a tobacco product including 32.3% in Djibouti, 39.4% in Mauritania, and 47.1% in Zimbabwe with a considerable proportion of the non-users being susceptible in some of these countries [3, 4]. In Gambia, a West African country, approximately 20% of adolescents reported ever using cigarettes while 25% of non-users were susceptible to smoking and over one-quarter had early initiation before 10 years of age [3], suggesting greater uptake of tobacco use among Gambian youth in recent years. Similarly, a study examining the prevalence of smoking among 10,289 students aged 12–20 years in Gambia found that the prevalence of ever smoking cigarettes, cigars, or pipes was 16.7%, with a higher incidence among boys (25.7%) than girls (9.4%) [5]. Current smoking (within the past 30 days preceding the survey) was 4.5%, with a higher prevalence among boys (7.9%) compared to girls (1.5%) [5]. Shisha smoking prevalence was unexpectedly high at 8.1%, particularly among girls (11.4% of boys and 5.4% of girls) [5]. Tobacco-related diseases are estimated to increase dramatically in the country and the entire SSA if current youth tobacco use trends continue [6]. These previous findings emphasise the importance of further research to better understand how young people in the Gambia are influenced by both pro and anti-tobacco media messages and events, and how this affects their smoking behaviour.

According to a study published in 2016, Gambia lacks a significant tobacco production sector and predominantly depends on imported tobacco products to satisfy domestic consumption needs. Particularly, the study reported that tobacco products supplied in the country are usually imported, with origins primarily from Senegal (39%), South Africa (22%), Swaziland (15%), Switzerland (14%), United Arab Emirates (6%), Nigeria (3%), the Netherlands (1%), and India (1%) [7]. Nevertheless, another study undertaken in 2004 found that free cigarette offers from tobacco company representatives were a significant factor influencing smoking behaviour in Gambia [8]. In 2012, a situational report published by the Gambian Ministry of Health and the African Network for Information and Action Against Drugs (RAID - The Gambia) indicated that despite the existence of tobacco use regulations in the country, tobacco companies continue to employ subtle advertising methods to promote their products in the country [9](The Gambian Ministry of Health and African Network for Information & Action Against Drugs, 2012). These methods include the use of shapes, films, vehicle colours, and containers to market tobacco products [9]. The study further noted that tobacco companies employed various marketing strategies across multiple platforms, including magazines, newspapers, television, radio, billboards, and the Internet. Brand stretching which involved selling or giving away non-tobacco products branded with a tobacco brand name and cigarette sampling were two common promotional tactics. For example, cigarette brand names may appear on T-shirts, hats, backpacks, and other items popular among adolescents, effectively turning the wearers into walking advertisements [9]. Price data shown in a recent study also indicates that approximately 7.3% of smokers bought illicit cigarettes during their last purchase and it was estimated that 8.6% of the total cigarette market comprised illicit products [10].

Historically, Gambia has implemented a series of legislative and policy measures to regulate tobacco use, starting with the Prohibition of Smoking (in Public Places) Act of 1998, which bans smoking in enclosed public spaces, workplaces, hospitals, public vehicles, and government premises [11]. In 2003, the Ban on Tobacco Advertisements Act was enacted, prohibiting the promotion of tobacco products in any form [11]. The country ratified the Framework Convention on Tobacco Control (FCTC) in 2007, followed by the introduction of Health Warning Directives in 2009, mandating that cigarette packs display warnings covering 30% of the principal display areas on both sides, including a “Sold in The Gambia” label [11]. In 2012, the Gambia established a National Tobacco Control Committee to formulate policies, and in 2013, implemented a three-year tobacco tax policy and a National Tobacco Control Policy and Action Plan (2013–2018) [11]. The Tobacco Control Act of 2016 was enacted to regulate tobacco products, alongside the launch of the National Clinical Guideline for Cessation Services in the same year [11]. Finally, in 2016, the Gambia acceded to the Illicit Trade Protocol, demonstrating its ongoing commitment to tobacco control efforts [11].

While there are various regulations intended to reduce smoking in the Gambia, exposure to tobacco advertisements remains high among adolescents in the country and several other SSA countries [12]. Evidence suggests that one potential factor influencing adolescent smoking behaviour is exposure to tobacco use messages, including anti-tobacco media campaigns, health warnings, tobacco advertising, and social norms [13]. Several studies have documented associations between exposure and receptivity to protobacco messages and youth smoking susceptibility, initiation, and intentions [13–17]. However, exposure to anti-tobacco messages has been linked to both increased [18] and reduced risk perceptions about smoking, lower intentions to smoke, and decreased smoking uptake in youthful populations [13, 19]. Surprisingly, limited research has examined the relationships between pro-tobacco and anti-tobacco media message exposure and smoking behaviour in youthful SSA populations [4]. Despite Gambia having a high prevalence of adolescent smoking, a high smoking susceptibility and an early smoking initiation before age 10 [3], limited published empirical studies on this topic have been identified in the country. A comprehensive exploration of these relationships is essential to gain a deeper understanding of the impact that exposure to tobacco-related media has on adolescent smoking behaviour within this specific region. Therefore, the primary aim of this paper is to examine associations between exposure to pro-tobacco and anti-tobacco media messages and events and smoking behaviour among a cohort of adolescents in Gambia using the 2017 Global Youth and Tobacco Survey (GYTS) data. Examination of these associations holds significant importance as it is key in shaping effective preventive measures. Understanding how exposure to different forms of tobacco-related media influences smoking behaviour among adolescents is critical for guiding targeted interventions and strategies aimed at tobacco use prevention. The insights derived from this study will contribute to a more robust comprehension of the impact of media exposure on adolescent tobacco use within the unique socio-cultural context of Gambia. Eventually, these findings are expected to provide valuable guidance for policymakers and public health initiatives aimed at curbing adolescent tobacco use in Gambia.

## Data and methods

### Data source

**Fig. 1:**
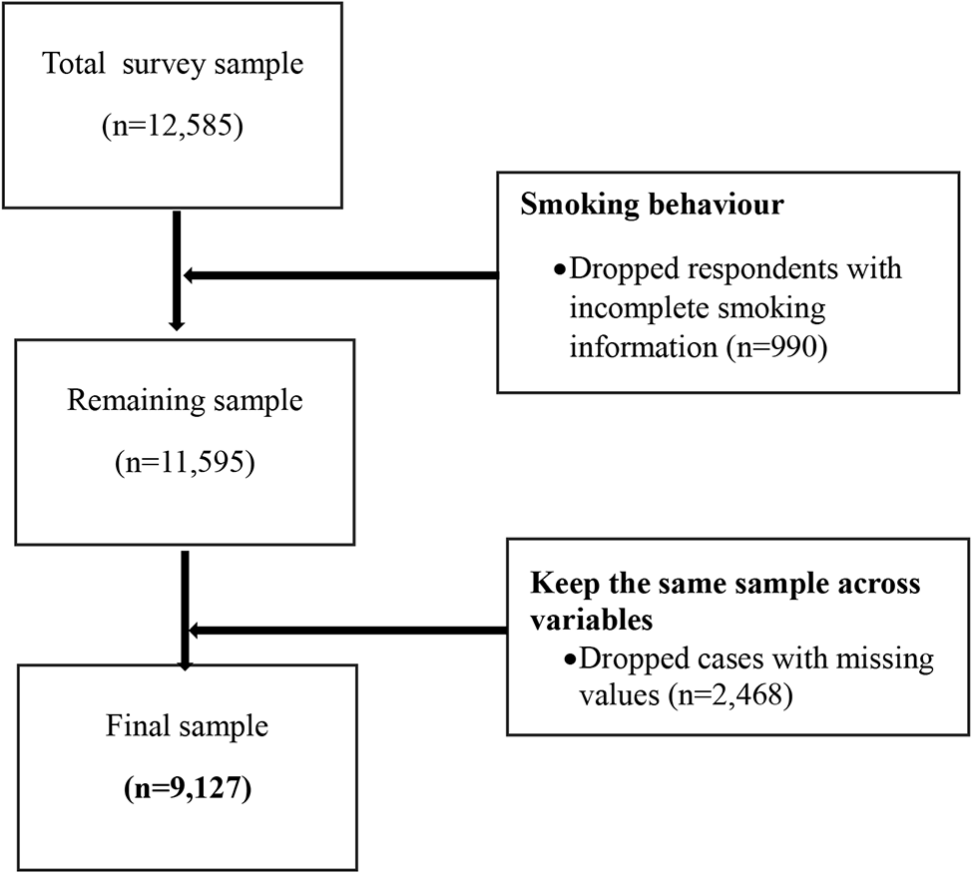
Flow chart of sample selection

We used the 2017 Global Youth and Tobacco Survey (GYTS) to examine the interplay of anti-tobacco media messages and events, pro-tobacco media messages, and smoking behaviour (outcome of interest) among adolescents aged 11–17 in Gambia. The GYTS is a widely known school-based survey developed by the Centers for Disease Control and Prevention (CDC) for the global monitoring of youth tobacco use and the guidance of tobacco prevention and control programs [20, 21]. The survey encompasses a representative cross-section of students in junior secondary schools (grades 7–9) and employs a two-stage cluster-sampling approach. In the first stage, schools are chosen with a probability proportional to their enrollment size, and in the second stage, classes within these schools are selected as a systematic equal probability sample with a random starting point [22]. All students in the selected classes were eligible to participate in the survey [20, 23]. The survey was conducted by the Ministry of Health and Social Welfare (MoHSW), with an overall response rate of 86.5% [21]. The 2017 GYTS initially covered a sample of 12,585 youths aged 11–17 years; however, after excluding cases with missing values, the final sample size used for the analysis was reduced to 9,127. (see Fig. 1). The survey employed dichotomised core questionnaires that included a set of optional questions. These questionnaires addressed various aspects related to tobacco use, such as smoking and smokeless tobacco, smoking cessation, exposure to secondhand smoke (SHS), both pro and anti-tobacco media and advertising, access to and availability of tobacco products, as well as knowledge and attitudes regarding tobacco use [24]. We relied on the Strengthening the Reporting of Observational Studies in Epidemiology (STROBE) guidelines in preparing this paper.

### Study variables and measurements

#### Outcome variable

The outcome of interest in this study is “smoking behaviour” which was defined as smoking in the past 30 days preceding the day of the survey. The response was dichotomised as ‘yes’=1 if a respondent met this criterion and ‘no’=0 if otherwise.

#### Main independent variables

The main independent variables used in this study were anti-tobacco messages and events and pro-tobacco messages (seeing people use tobacco on television, seeing tobacco advertisements, and the respondent owning *an object with a tobacco brand logo*). These variables were measured dichotomously with “yes = 1” and “no = 0” responses.

#### Covariates

**Fig. 2:**
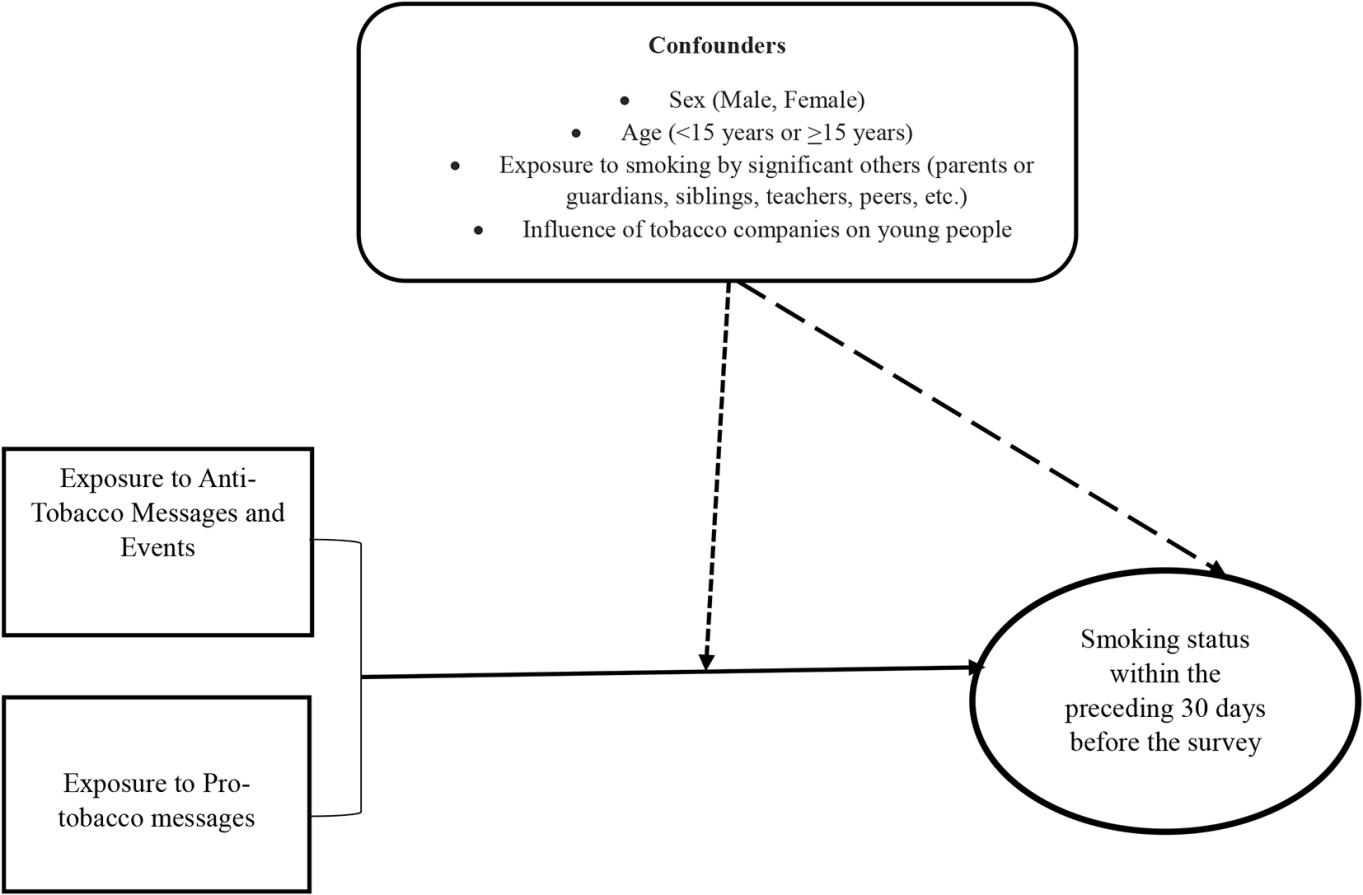
A causal diagram showing the study variables and confounders

A number of potential confounding explanatory variables were included as covariates. These variables include sex (male and female), age (less than 15 years, and 15 and above); exposure to smoking behaviour by significant others including parents, closest friend, mother smoking at home, father smoking at home, brother/sister smoking at home, other people smoking at home, people smoking in school, teachers smoking in the school building; and tobacco companies influencing young people to use tobacco (see Fig. 2).

In the diagram, the main independent variables (anti-tobacco messages and events, and pro-tobacco messages) are shown on the left side, influencing the outcome of interest or dependent variable (smoking behaviour) on the right side. Confounding variables such as sex, age, and exposure to smoking behaviour by significant others, as well as the influence of tobacco companies on young people, are included as covariates that may affect the relationship between the independent and dependent variables, indicated by a short dotted line. Also, the confounders may have an indirect effect on the outcome variable, independent of their influence on the main independent variables as shown by the long dotted line.

### Statistical analysis

The study employed both descriptive and inferential analyses. Descriptively, we estimated the proportion of children currently using tobacco based on their sociodemographic characteristics. To assess differences among these proportions, we employed Pearson’s chi-squared test. For the multivariable analysis, we employed a logistic regression model to estimate adjusted odds ratios and 95% confidence intervals. This allowed us to examine the associations between exposure to anti-tobacco messages and events, exposure to pro-tobacco messages, and smoking behaviour while adjusting for relevant covariates. Four multivariable logistic regression models were fitted, with Model I including only the main independent variables while Model II adjusted for the sex and age of the respondents. Model III adjusted for the variables related to exposure to smoking behaviour by significant others and the final model (Model IV) adjusted for all relevant covariates considered in this study. Akaike’s Information Criterion (AIC) was employed to assess the fitness of the models. The model characterised by the lowest value of the information criterion was chosen as the best model in the analysis. A multicollinearity test with a mean-variance inflation factor (VIF) of 8.54 was observed for the analysis. The analysis was sample-weighted to cater for over-sampling and under-sampling concerns in the data. All statistical analyses were performed with Stata Version 17.0.

## Results

### Descriptive results

Of the 9,127 participants, 727 (8.0%) reported engaging in smoking. Notably, those exposed to anti-tobacco media messages demonstrated a higher smoking rate of 9.3% compared to individuals not exposed to such messages (6.9%). Conversely, participants who were not exposed to anti-tobacco media events displayed a higher smoking prevalence of 11.3% in contrast to those exposed (6.9%).

Regarding exposure to pro-tobacco messages, respondents who witnessed people using tobacco on television exhibited a notably higher smoking rate of 10.0% compared to those who did not encounter such messages (6.0%). Similarly, those who saw tobacco advertisements showed a higher prevalence of smoking, recording 11.4% in contrast to individuals not exposed to these ads (7.4%). Moreover, respondents owning objects with tobacco brand logos had a significantly higher smoking rate of 14.2% compared to those without such items (7.3%).

Regarding gender differences, male respondents demonstrated a substantially higher smoking prevalence of 13.9% compared to their female counterparts, with a smoking rate of 2.9%. Additionally, older respondents aged 15 years and above displayed a higher smoking proportion compared to those below 15 years.

Furthermore, respondents with both parents smoking tobacco showed a significantly higher smoking rate of 15.7% compared to individuals whose parents did not smoke (6.6%). Similarly, participants who reported that most of their friends smoke displayed a strikingly higher smoking prevalence of 20.1% compared to those with none of their friends smoking (4.7%).

The study also revealed higher smoking rates among individuals whose mothers (15.7%) or fathers (14.0%) occasionally smoked at home compared to those with non-smoking parents at home (mothers: 6.8%, fathers: 6.1%). Likewise, respondents with siblings occasionally smoking at home showed a notably higher smoking rate of 18.6% compared to those whose siblings did not smoke at home (6.4%).

Finally, participants who reported that their teachers smoke in school buildings almost every day had a higher smoking prevalence, recording 12.6%. A detailed breakdown of the smoking prevalence among these respondents based on various background characteristics is presented in Table 1.

**Table 1:** Distribution of adolescent smokers by background characteristics

Explanatory variables	Weighted frequencies [n (%)]	Proportion smoking		*p*-value
		(n)	(%)	
**Expose to anti-tobacco media messages**				< 0.001
*Yes*	3,888(42.6)	363	9.3	
*No*	5,238(57.4)	364	6.9	
**Expose to anti-tobacco media events**				< 0.001
*Yes*	2,252(24.7)	253	11.3	
*No*	6,875(75.3)	474	6.9	
**Expose to pro-tobacco messages**				
**See people using tobacco on TV**				< 0.001
*Yes*	4,536 (49.7)	453	10.0	
*No*	4,590 (50.3)	274	6.0	
**See tobacco advertisements**				< 0.001
*Yes*	1,296(14.2)	148	11.4	
*No*	7,831(85.8)	579	7.4	
**Ownership of an object with a tobacco brand logo**				< 0.001
*Yes*	869(9.5)	123	14.2	
*No*	8,258(90.5)	604	7.3	
**Sex**				< 0.001
*Male*	4,203(46.01)	582	13.9	
*Female*	4,924(53.9)	145	2.9	
**Age**				< 0.001
*< 15 years*	3,873(42.4)	235	6.1	
*15 years and above*	5,254(57.6)	492	9.4	
**Grade/form**				0.001
*7*	3,666(35.8)	210	6.4	
*8*	2,002(32.9)	268	8.9	
*9*	2,858(31.3)	249	8.7	
**Parents smoke**				< 0.001
*None*	7,220(79.1)	475	6.6	
*Both*	181(2.0)	28	15.7	
*Father only*	932(10.2)	150	16.1	
*Mother only*	268(2.9)	31	11.4	
*Don’t know*	526(5.8)	43	8.2	
**Closest friend smoke**				< 0.001
*None of them*	7,136(78.2)	336	4.7	
*Some of them*	1,568(17.2)	317	20.2	
*Most of them*	302(3.3)	61	20.1	
*All of them*	121(1.3)	13	11.0	
**Mother smoking in your home**				< 0.001
*Don’t have/see this person*	3,337(36.6)	252	7.5	
*About every day*	332(3.6)	49	14.7	
*Sometimes*	600(6.6)	94	15.7	
*Never*	4,857(53.2)	332	6.8	
**Father smoking in your home**				< 0.001
*Don’t have/see this person*	3,630(39.8)	251	7.0	
*About every day*	690(7.6)	91	13.2	
*Sometimes*	1,132(12.4)	159	14.0	
*Never*	3,675(40.3)	226	6.1	
**Brother/sister smoking in your home**				< 0.001
*Don’t have/see this person*	3,127(34.3)	218	7.0	
*About every day*	383(4.2)	58	15.1	
*Sometimes*	749(8.2)	139	18.6	
*Never*	4,867(53.3)	312	6.4	
**Other people smoking in your home**				< 0.001
*Don’t have/see this person*	2,072(22.7)	130	6.3	
*About every day*	814(8.9)	93	11.5	
*Sometimes*	3,049(33.4)	360	11.8	
*Never*	3,192(35.0)	144	4.5	
**People smoke in school**				< 0.001
*Yes*	2,609(28.6)	310	11.9	
*No*	6,517(71.4)	417	6.4	
**Teachers smoking in school buildings**				< 0.001
*About every day*	543(5.91)	68	12.6	
*Sometimes*	1,725(19.00)	211	12.2	
*Never*	5,085(55.67)	338	6.6	
*Don’t know*	1,774(19.41)	109	6.2	
**Tobacco companies influence young people to use tobacco**				**0.005**
*Yes*	3,076 (33.7)	282	9.2	
*No*	6,050(66.3)	445	7.4	
**Total**	**9,127**	**727**	**8.0**	

*Notes* “n” represents the sample size or the total number of observations, % represents percentage, and the p-value represents the significant levels; p-values are based on Chi-square test

### Multivariable logistic regression on the interplay of exposure to pro-tobacco and anti-tobacco media messages and events, and smoking behaviour

As shown in Table 2, there were significant associations between various factors and smoking behaviour among the respondents. Exposure to anti-tobacco media messages and events, witnessing tobacco use on TV, owning objects featuring tobacco brand logos, as well as sex, age, parental smoking, influence of closest friends and family members smoking at home, exposure to smoking in school environments, specifically teachers smoking in school buildings, emerged as notably linked to smoking behaviour. Specifically, respondents exposed to anti-tobacco media messages displayed higher odds of smoking (aOR = 1.29, CI = 1.08,1.53) compared to those unexposed. Similarly, those exposed to anti-tobacco media events showed increased odds of smoking (aOR = 1.31, CI = 1.09, 1.59) compared to their non-exposed counterparts. Those who observed people using tobacco on television were more likely to smoke (aOR = 1.41, CI = 1.17, 1.69) compared to those who did not witness such portrayals.

Ownership of an object displaying a tobacco brand logo was associated with higher odds of smoking (aOR = 1.49, CI = 1.19,1.86) in comparison to those without such possessions. Additionally, male respondents demonstrated substantially higher odds of smoking (aOR = 4.01, CI = 3.28,4.89) compared to their female counterparts.

Age was also a significant factor, as respondents aged 15 years and older showed increased odds of smoking (aOR = 1.47, CI = 1.22,1.76) compared to those below 15 years old. Furthermore, respondents with only their father smoking displayed higher odds of smoking (aOR = 1.35, CI = 1.04,1.76) compared to individuals with non-smoking parents.

Among other influential factors, respondents with some of their closest friends smoking demonstrated notably higher odds of smoking (aOR = 2.87, CI = 2.37, 3.48) compared to those without such influence. Similarly, those with family members, specifically fathers or siblings, occasionally smoking at home showed increased odds of smoking (Father: aOR = 1.71, CI = 1.29,2.28; Brother/Sister: aOR = 1.61, CI = 1.22,2.13) compared to individuals without these household influences.

Additionally, respondents who reported teachers smoking in school buildings almost daily showed higher odds of smoking (aOR = 1.38, CI = 1.02,1.87) compared to those who did not encounter such occurrences in school. These findings highlight the multifaceted associations between diverse factors and smoking behaviour among the studied population.

**Table 2:** Multivariable logistic regression on the interplay of exposure to pro-tobacco and anti-tobacco media messages and events, and smoking behaviour

Explanatory variables	Model 1	Model 2	Model 3	Model 4 (Final model)
	Crude odds ratio (COR)	Adjusted odds ratio (aOR)	Adjusted odds ratio (aOR)	Adjusted odds ratio (aOR)
**Expose to anti-tobacco media messages**				
*Yes*	1.35***[1.14,1.60]			1.29**[1.08,1.53]
*No*	1.00			1.00
**Expose to anti-tobacco media events**				
*Yes*	1.49***[1.25,1.78]			1.31**[1.09,1.59]
*No*	1.00			1.00
**Expose to pro-tobacco messages**				
See people using tobacco on TV				
*Yes*	1.69***[1.43,2.00]			1.41***[1.17,1.69]
*No*	1.00			1.00
See tobacco advertisements				
*Yes*	1.24*[1.01,1.53]			1.11[0.89,1.39]
*No*	1.00			1.00
Ownership of an object with a tobacco brand logo				
*Yes*	1.99***[1.62,2.45]			1.49**[1.19,1.86]
*No*	1.00			1.00
**Covariates**				
Sex				
*Male*		5.21***[4.30,6.32]		4.01***[3.28,4.89]
*Female*		1.00		1.00
*Age*				
*< 15 years*		1.00		1.00
*15 years and above*		1.57***[1.32,1.86]		1.47***[1.22,1.76]
**Exposure to smoking behaviour by significant others**				
Parents smoke				
*None*			1.00	1.00
*Both*			1.13[0.70,1.81]	1.14[0.71,1.85]
*Father only*			1.30*[1.01,1.68]	1.35*[1.04,1.76]
*Mother only*			1.35[0.99,2.06]	1.47[0.96,2.26]
*Don’t know*			0.89[0.62,1.28]	0.93[0.64,1.35]
Closest friend smoke				
*None of them*			1.00	1.00
*Some of them*			4.26***[3.55,5.12]	2.87***[2.37,3.48]
*Most of them*			3.36***[2.37,4.77]	2.46***[1.72,3.51]
*All of them*			3.15***[1.94,5.10]	2.67***[1.61,4.43]
Mother smoking in your home				
*Don’t have/see this person*			0.98[0.76,1.25]	0.93[0.72,1.20]
*About every day*			1.26[0.86,1.83]	1.42[0.96,2.09]
*Sometimes*			1.03[0.75,1.40]	1.06[0.77,1.46]
*Never*			1.00	1.00
Father smoking in your home				
*Don’t have/see this person*			1.26[0.98,1.61]	1.27[0.99,1.64]
*About every day*			1.38[0.99,1.93]	1.38[0.97,1.97]
*Sometimes*			1.74***[1.33,2.29]	1.71***[1.29,2.28]
*Never*			1.00	1.00
Brother/sister smoking in your home				
*Don’t have/see this person*			0.73*[0.57,0.94]	0.75*[0.58,0.96]
*About every day*			1.34[0.95,1.89]	1.26[0.97,1.97]
*Sometimes*			1.71***[1.30,2.23]	1.61**[1.22,2.13]
*Never*			1.00	1.00
Other people smoking in your home				
*Don’t have/see this person*			1.39*[1.03,1.87]	1.37*[1.01,1.87]
*About every day*			1.64**[1.19,2.25]	1.70**[1.22,2.36]
*Sometimes*			1.84***[1.45,2.34]	1.82***[1.42,2.32]
*Never*			1.00	1.00
People smoke in school				
*Yes*			1.52***[1.27,1.83]	1.42***[1.17,1.72]
*No*			1.00	1.00
Teachers smoking in school buildings				
*About every day*			1.54**[1.15,2.05]	1.38*[1.02,1.87]
*Sometimes*			1.35**[1.09,1.68]	1.31*[1.05,1.64]
*Don’t know*			0.88[0.68,1.14]	0.92[0.71,1.20]
*Never*			1.00	1.00
**Tobacco companies influence young people to use tobacco**				
*Yes*			1.13[0.95,1.34]	1.16[0.97,1.39]
*No*			1.00	1.00
*Constant*	0.04***[0.03,0.04]	0.02***[0.02,0.03]	0.02***[0.01,0.02]	0.00***[0.00,0.01]
**Model fitness**				
*Prob* > *Chi*^2^	< 0.001	< 0.001	< 0.001	< 0.001
*AIC*	4504.184	4264.787	4118.502	3832.684

1.00: reference category; AIC: Akaike Information Criterion ***p < 0.001, **p < 0.010, *p < 0.050

## Discussion

This study aimed to investigate the relationships between exposure to pro and anti-tobacco media messages and events, and the smoking behaviour of adolescents (11–17 years) in Gambia, a West African country, characterised by a notably high incidence of adolescent smoking [4, 25]. The use of multivariable logistic regression in assessing this objective has facilitated a comprehensive understanding of the various factors significantly associated with smoking behaviour within this adolescent demographic, providing invaluable insights essential for addressing adolescent smoking behaviour in Gambia.

Primarily, the findings highlight a substantial influence of media exposure and the social environment on adolescent smoking behaviour in Gambia. Specifically, exposure to anti-tobacco media messages and anti-tobacco media events were both associated with elevated odds of smoking among adolescents, demonstrating a 29% and 31% higher likelihood of smoking, respectively, when compared to their non-exposed counterparts. Similarly, exposure to pro-tobacco messages, involving instances such as encountering tobacco use on television or possessing items decorated with tobacco brand logos, was linked to a remarkably higher increase in smoking prevalence, demonstrating around 41% elevated likelihood among adolescents. These results collectively suggest a concerning relationship between exposure to anti-tobacco and pro-tobacco messaging with adolescent smoking behaviour, underscoring the complex and influential role that the media plays in shaping youth smoking habits, with pro-tobacco messages potentially exerting a more substantial impact on smoking prevalence compared to anti-tobacco messages and events. In line with our findings, it is not surprising that a review study found anti-smoking advertising to be less effective in several contexts partly due to the influence of pro-tobacco advertising and marketing [26]. Potential explanations for these outcomes could revolve around several factors. Firstly, while exposure to anti-tobacco messages may effectively reduce adolescent smoking behaviour in various instances [13, 27], it can also unintentionally spark curiosity through repeated exposure, whereas exposure to pro-tobacco messages can normalise smoking in adolescents leading some adolescents to explore smoking [28–30]. This curiosity might be driven by a desire to understand the discouraged or promoted behaviour or to validate the severity of the warnings [25, 31]. Moreover, the messages or events advocating against tobacco use may lack comprehensive information, failing to effectively communicate the negative consequences of smoking [32]. Adolescents exposed to incomplete messages may not fully grasp the severity of the risks associated with smoking, thus underestimating its harm [33]. From a methodological perspective, these findings may also reflect the complexities of human behaviour and the limitations inherent in correlation-based studies. While the present study indicates a connection between exposure to anti-tobacco media and an increased likelihood of smoking, it is essential to be reminded that correlation does not equate to causation [34]. Other unaccounted variables or individual predispositions could influence both exposure to anti-tobacco media and smoking behaviour. For example, broader contextual factors, such as the content and tone of the message, an individual’s susceptibility, or concurrent social influences could be contributing factors to these findings [35–37]. This suggests the need for further research, involving detailed longitudinal and qualitative investigations to understand the complex connections between exposure to pro-tobacco and anti-tobacco media and adolescent smoking behaviour.

In addition to media exposure, the present findings emphasise the significant influence of familial and peer smoking behaviour on adolescents. Smoking among parents, closest friends, and household members showed strong associations with increased odds of adolescent smoking. Particularly, adolescents who had both parents smoking constituted higher smoking prevalence. There are several plausible reasons for this finding. First, adolescents commonly tend to simulate the conduct of their parents, particularly in the context of smoking behaviour [38]. Habitual smoking practices of both parents can establish a pattern of normalcy within the household environment [39]. Consequently, this normalisation of smoking can create a perception among adolescents that smoking is socially acceptable and endorse its adoption as part of their behaviour [38, 39]. Furthermore, the presence of parents engaging in smoking can be interpreted as an implicit endorsement within the family unit [40]. This parental conduct serves to validate smoking as a customary and permissible practice, thereby influencing adolescents to potentially embrace smoking as an accepted behaviour [40]. Moreover, parents engaging in smoking behaviour can amplify the accessibility of tobacco products within the household, facilitating adolescents’ access to tobacco and potentially encouraging experimentation with smoking [41, 42].

Similarly, peer smoking behaviour displayed a graded association with increased smoking prevalence in this present study, emphasising the substantial impact of peer groups on adolescent smoking behaviour. This finding suggests that as smoking behaviour among adolescent peers intensifies, there is a potential corresponding increase in the prevalence of smoking within that particular social group or community. This may be due to the point that adolescents are highly susceptible to peer influence and the desire to fit in or be accepted within their social circles, can influence them to engage in behaviour they might otherwise be hesitant to perform [42–44]. Thus, if smoking is perceived as a norm or a socially accepted behaviour among their peers, adolescents may feel pressured to imitate the behaviour to conform to group norms and gain social acceptance [45]. In line with our findings, a meta-analysis study has indicated a remarkable increase in adolescent smoking behaviour attributed to both peers and family members [46]. These findings underscore the critical need for interventions aimed at reshaping social norms regarding smoking within familial and peer networks as these could significantly affect adolescent smoking rates.

Besides the key findings discussed thus far, covariates such as gender and age presented significant associations with adolescent smoking behaviour, with males having higher odds of smoking compared to females. Potential explanations underlie an observed trend wherein smoking often aligns with greater acceptability and anticipation among males within several traditional African societies [47, 48]. Moreover, historical marketing strategies by tobacco companies have specifically targeted males through promotional campaigns that associate smoking with traits such as ‘masculinity’, ‘adventure’, or ‘toughness’ [49, 50]. Such tailored marketing approaches might exert a more pronounced influence on males, potentially prompting them to initiate or sustain smoking behaviour. Additionally, smoking might serve as a coping mechanism for males in certain instances, offering a means to address stress or navigate emotional challenges [51, 52]. Thus, societal expectations that emphasise the suppression of emotions among men could lead to the adoption of smoking as a method for alleviating stress, thereby contributing to the higher prevalence of smoking among males compared to females [51, 52].

Furthermore, older adolescents (15 years and above) displayed increased odds of smoking. Numerous contributing factors may explain this finding. As adolescents progress into older age brackets, their social spheres are likely to expand, introducing them to peer groups or social contexts where smoking may prevail more prominently [53, 54]. The increased exposure to peers who smoke or environments, where smoking is prevalent, holds the potential to exert influence on older adolescents, instigating the initiation or continuation of smoking practices [53, 54]. Additionally, the progression into older adolescence coincides with a phase marked by amplified curiosity and an ‘appetite’ for experimentation [25]. This developmental stage prompts older adolescents to demonstrate a greater inclination toward smoking, driven by growing independence and a quest for experiences beyond their immediate familial boundaries [25, 53]. Moreover, older adolescents may develop an increased self-perception of maturity and invincibility to risks [55]. This perception, coupled with the characteristic inclination toward sensation-seeking behaviour common during adolescence may contribute to a heightened willingness to partake in behaviour perceived as risky, such as smoking [56]. These findings reveal the importance of targeted anti-tobacco campaigns and the need for interventions addressing diverse influences to mitigate adolescent smoking prevalence. Implementing comprehensive anti-tobacco media campaigns targeted specifically at varying age groups and genders could yield substantial benefits in reducing smoking prevalence among adolescents. Moreover, imposing stringent regulations on tobacco advertisements and reducing the visibility of tobacco use on media platforms can be used as pivotal strategies to counter smoking initiation among adolescents. Strengthening policies to enforce restrictions on tobacco marketing, particularly concerning items branded with tobacco logos, becomes imperative in light of this study’s findings. Providing immediate smoking cessation support for young people through effective services is also crucial. This is particularly important considering a previous study indicating that despite a majority of smokers expressing a desire to quit, only 22% reported receiving cessation support in Gambia [5].

The findings also suggest that tobacco companies have a role in influencing young people to use tobacco. This is supported by previous studies indicating that tobacco marketing strategies are associated with tobacco use initiation among adolescents. For example, a previous study found that free cigarette offers from tobacco company representatives were a significant factor in recognising smokers from non-smokers [8]. Likewise, the Gambian Ministry of Health and the African Network for Information & Action Against Drugs have reported the use of subtle advertising methods by tobacco companies, such as shapes, films, vehicle colours, and containers, to market tobacco products [9]. The report suggested that the 2003 Tobacco Control Act prohibits the advertisement of tobacco products in various forms, encompassing print and electronic media, as well as other modes of communication [9]. However, a significant limitation of the act lies in its lack of clarity on what constitutes advertisement or promotion. This ambiguity has provided tobacco companies with some latitude to engage in advertising practices in secret. These tactics indicate a deliberate effort by tobacco industries to target and influence adolescents, potentially contributing to the uptake of smoking in this demographic. The persistence of these marketing strategies despite efforts to curb smoking initiation underscores the need for continued vigilance and regulatory action to protect young people from tobacco industry influence [9].

For future research, evaluating the effectiveness of culturally tailored anti-tobacco media campaigns and conducting longitudinal or qualitative studies to track the long-term impact of media exposure on smoking behaviour could provide deeper insights. Further exploration of additional contributing factors beyond media influence would also enrich the understanding of determinants shaping adolescent smoking behaviour in this understudied population.

### Strengths and limitations

This study contributes significantly to tobacco control research by shedding light on an area that has been relatively under-examined in Gambia. The inclusion of nationally representative data is a notable strength, enhancing the study’s scope and lending credibility to the findings. The study’s outcomes also serve as a valuable estimative tool at the national level, providing insights into the magnitude of adolescent smoking issues in the country. Moreover, the findings establish a foundational platform for future research, allowing for deeper investigations into various facets of anti-smoking campaigns, including message content and program effectiveness, thereby fostering more comprehensive and in-depth analyses in the future.

Despite the strengths of this study, some limitations need acknowledgement. First, the study’s reliance on a school-based survey restricts the representation of adolescents solely to those attending school, potentially excluding out-of-school adolescents and those in alternative educational settings. This limitation may hinder a comprehensive understanding of smoking use among all adolescents, leading to potential biases in prevalence estimates. Second, the use of self-reported responses is susceptible to recall and social desirability bias, raising concerns about data accuracy and reliability due to subjective participant responses. Third, the absence of contextual factors, such as national tobacco control plans in the analysis due to unavailability in the datasets, limits a more comprehensive understanding of the factors shaping adolescent smoking behaviour. Lastly, the cross-sectional nature of the GYTS data poses another limitation as it prevents the establishment of causal relationships between exposure to tobacco-related messages and actual smoking behaviour among adolescents.

Addressing the limitations of the 2017 Gambia Global Youth and Tobacco Survey proved challenging due to the secondary nature of the data. Nevertheless, data validity and reliability were ensured through several strategies. Firstly, to enhance the validity of the findings, missing values such as no responses were first excluded from the analysis. This step is essential as missing data can lead to biased results and reduce the accuracy of the findings. By excluding these values, the analysis focused only on complete and reliable data, ensuring that the results are more representative of the sample and less prone to errors. Secondly, the analysis was sample-weighted to account for over-sampling and under-sampling issues, ensuring the results were representative of the population under study. Additionally, the final model (Model IV) was adjusted for all relevant covariates, minimising the potential impact of confounding variables. Akaike’s Information Criterion (AIC) was utilised to assess the fitness of the models, with the model exhibiting the lowest AIC value selected as the best fit. To address multicollinearity concerns, a test was conducted, revealing a mean-variance inflation factor (VIF) of 8.54, which was within acceptable limits. Despite the potential for self-reporting bias, the survey employed validated questionnaires that were piloted to enhance their accuracy. To improve future studies, however, we recommend conducting community-based surveys or outreach programs to encompass out-of-school adolescents and those in alternative educational settings. Furthermore, we advocate for future longitudinal studies to establish causal relationships between exposure to tobacco-related messages and actual smoking behaviour among adolescents.

## Conclusion

Based on the findings, we conclude that exposure to anti-tobacco media messages and events has a notable association with increased odds of smoking. Adolescents exposed to anti-tobacco media messages demonstrated a 29% higher likelihood of smoking, while exposure to anti-tobacco media events was associated with a 31% higher likelihood of smoking. Similarly, exposure to pro-tobacco messages showed a substantial positive association with increased odds of smoking initiation among adolescents. Witnessing individuals using tobacco on TV was associated with a 41% higher likelihood of smoking, while ownership of items featuring tobacco brand logos showed a 49% higher likelihood of smoking. These findings emphasise the detrimental impact of pro-tobacco media exposure on adolescent smoking behaviour.

Moreover, significant associations were observed between the smoking behaviour of significant others and adolescent smoking prevalence. Parental smoking exhibited an increased likelihood of adolescent smoking. Correspondingly, the smoking behaviour of closest friends displayed graded associations; having some or most friends who smoke led to significantly increased odds of adolescent smoking. Overall, this study emphasises the significant impact of diverse factors, particularly exposure to both anti-tobacco and pro-tobacco media messages, as well as the influence of significant others’ smoking behaviour and school environments, in shaping adolescent smoking behaviour. These findings hold substantial implications for research, practice, and policy in adolescent health and tobacco control.

## Data Availability

The data are publicly available in an open-access repository: Data are publicly available on: https://nccd.cdc.gov/GTSSDataSurveyResources/Ancillary/DataReports.aspx?CAID=2.

## Abbreviations

AIC: Akaike’s Information Criterion
AOR: Adjusted Odds Ratios
CI: Confidence Interval
COR: Crude Odds Ratio
FCTC: Framework Convention on Tobacco Control
GYTS: Gambia Global Youth and Tobacco Survey
MoHSW: Ministry of Health and Social Welfare
OR: Odds Ratio
SSA: Sub-Saharan Africa
STROBE: Strengthening the Reporting of Observational Studies in Epidemiology
TV: Television
VIF: Variance Inflation Factor
